# Assessment of the Effectiveness of a Seasonal-Long Insecticide-Based Control Strategy against *Aedes albopictus* Nuisance in an Urban Area

**DOI:** 10.1371/journal.pntd.0004463

**Published:** 2016-03-03

**Authors:** Beniamino Caputo, Mattia Manica, Antonello D’Alessandro, Giordano Bottà, Federico Filipponi, Carmela Protano, Matteo Vitali, Roberto Rosà, Alessandra della Torre

**Affiliations:** 1 Dipartimento di Sanità Pubblica e Malattie Infettive, Istituto Pasteur–Fondazione Cenci Bolognetti, Università di Roma “Sapienza”, Rome, Italy; 2 Dipartimento di Biodiversità ed Ecologia Molecolare, Centro Ricerca e Innovazione, Fondazione Edmund Mach, San Michele all'Adige, Trentino, Italia; Mahidol University, THAILAND

## Abstract

Seasonal-long larvicide treatments and/or outdoor space-spray applications of insecticides are frequently applied to reduce *Aedes albopictus* nuisance in urban areas of temperate regions, where the species has become a permanent pest affecting people’s quality of life and health. However, assessments of the effectiveness of sequential interventions is a difficult task, as it requires to take into account the cumulative and combined effect of multiple treatments, as well as the mosquito seasonal dynamics (rather than mosquito abundance before and after single treatments). We here present the results of the effectiveness assessment of a seasonal-long calendar-based control intervention integrating larvicide treatments of street catch basins and night-time adulticide ground spraying in the main University hospital in Rome (Italy). Cage-experiments and an intensive monitoring of wild mosquito abundance in treated and untreated sites were carried out along an entire season. Sticky traps were used to monitor adult abundance and site-specific eco-climatic variations (by recording water left over in each trap), in order to disentangle the effect of insecticide treatments from eco-climatic drivers on mosquito seasonal dynamics. Despite the apparent limited impact of single adulticide sprayings assessed based on mortality in caged and wild mosquitoes, the results of the temporal analysis showed that mosquito seasonal patterns were initially comparable in the two sites, diverged in the absence of diverging eco-climatic conditions and remained stable afterwards. This allowed to attribute the lack of the expected *Ae*. *albopictus* population expansion in the treated site to the combined effect of multiple adulticide sprayings and larvicide treatments carried out during the whole season. The approach proposed was proved to be successful to assess effects of seasonal-long control treatments on adult mosquito population dynamics and could represent a valuable instrument to assess the effectiveness of other control interventions, to evaluate their actual cost-benefits and to possibly minimize space-spraying applications to reduce mosquito nuisance.

## Author Summary

Due to the considerable nuisance created by the aggressive day-time biting behaviour of the “tiger mosquito,” insecticide treatments are largely employed in urban areas of temperate regions where this tropical species has become a permanent pest. These include treatments of catch basins with lethal products for mosquito larvae and spraying of insecticides against flying adults. Despite the latter having a high environmental impact and not being recommended by health authorities to reduce the nuisance to citizens, they are perceived as the approach providing the greatest benefits. However, this is not fully demonstrated. We showed that a seasonal-long calendar-based control intervention carried out against mosquito larvae and adults in Rome (Italy) was effective in reducing the mosquito abundance in the months when highest densities and nuisance are known to occur. The novel methodological approach followed here facilitates the assessment of the actual effectiveness of control strategies against mosquitoes, which are very rarely assessed due to technical difficulties, high costs and lack of commitments, but are instrumental to optimize control strategies. Should our preliminary indications of a major effect of larvicide treatments be confirmed, the more harmful exploitation of insecticide spraying could be reduced or eliminated.

## Introduction

In the case of major malaria and Dengue vector species, which are the most frequent targets of insecticide-based interventions, the most important parameter to define the effectiveness of a treatment is its impact on disease transmission and morbidity/mortality. In the absence of disease transmission, standardized methodological and statistical approaches and guidelines to assess the effectiveness of insecticides against mosquitoes mostly focus on the assessment of the effectiveness of single treatments [1,2]. In the case of adulticide treatments, this is carried out by measuring either mortality in caged mosquitoes spread in the target area, or percentages of reduction in wild mosquito abundance between pre- and post-treatment (e.g. by Abbot and Henderson’s formula),taking into account technical aspects (e.g. insecticide product, droplet size, time and length of spraying) and meteorological conditions (e.g. wind, temperature).

Assessments of the effectiveness of sequential insecticide-based interventions is a more difficult task, as it requires to take into account the cumulative and combined effects of multiple treatments, as well as the mosquito seasonal dynamics, rather than mosquito abundance only. Moreover, in order to compare mosquito populations over time it is recommended that similar paired sites (treated and untreated) are selected according to mosquito population parameters (e.g. density, population dynamics, isolation), as well as socio-economic, climatic and ecological (e.g. landscape, availability of breeding sites, presence of competing species) factors[3,4]. Ideally, in order to provide significant preliminary data, the two sites should be selected and monitored along the mosquito reproductive season before the treatments or at least a few weeks beforehand. This exercise is laborious and costly, and even if results show similar vector densities and dynamics, eco-climatic changes occurring in one of the two sites may interfere with the subsequent assessment of the effectiveness of seasonal long control interventions.

Seasonal-long outdoor space-spray applications of insecticides, integrated or not with other mosquito control activities, are frequently applied to reduce *Aedes albopictus* nuisance in urban areas in temperate regions. In fact, this originally Asiatic tropical species has become a permanent pest and is affecting citizen’s quality of life and health [5] in US and Europe since its introduction in the ‘80 [6,7] and ‘90[8,9], respectively. Due to above mentioned constraints, only limited field assessments of seasonal-long area-wide strategies to reduce *Ae*. *albopictus* densities (and nuisance) have been carried out so far. Source reduction campaigns were shown to achieve temporary suppression of immature *Ae*. *albopictus* in Spain [10] and in North Carolina [11], but they were not sufficient to maintain adult counts below a nuisance threshold in New Jersey[12]. In the latter work, Fonseca et al. also showed that integrated area-wide control strategies (i.e. active source reduction, larviciding, adulticiding and public education) resulted in a substantial reduction in *Ae*. *albopictus* populations in urban sites but not in suburban ones [12].

In Italy—where *Ae*. *albopictus* represents a major pest in urban and periurban areas and has already been responsible of a chikungunya virus outbreak [13]—seasonal-long outdoor interventions are frequently carried out to control its nuisance either in public or private urban areas. These interventions include multiple sequential larvicide treatments of street catch basins (considered the major not-removable urban larval sites [14,15]) and/or outdoor cold fog adulticide applications using vehicle-mounted sprayers. Data by Caputo et al.[14] suggest that the major phase of *Ae*. *albopictus* population expansion in Rome may be prevented by seasonal-long larvicide treatments of street catch basins in association with adulticide sprayings carried out during sunset.

We here present the results of the assessment of the effectiveness of a seasonal-long calendar-based control intervention integrating larvicide treatments of street catch basins and night-time adulticide ground spraying against *Ae*. *albopictus* in the main University hospital in Rome. Cage-experiments and a fine-scale monitoring of wild mosquito abundance in the study site were carried out along an entire season. At the same time, an *ad hoc* developed easy-to-use approach was implemented to measure micro eco-climatic changes in treated and control sites. Results were exploited to assess the effectiveness of single adulticide treatments on mosquito abundance before and after single sprayings, as well as the overall effectiveness of the integrated intervention on the mosquito population dynamics.

## Methods

### Study sites

**Fig 1:**
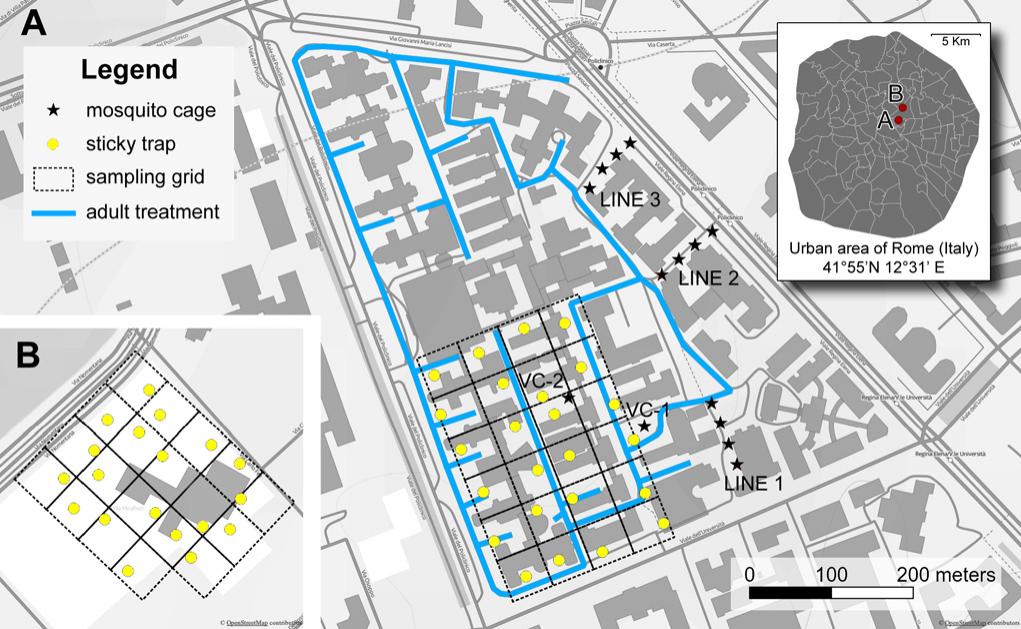
Map of study sites in Rome (Italy). (A) Sapienza University hospital “Policlinico Umberto I” = insecticide treated site (right panel); dark grey = buildings; light grey = open areas; blue line = itinerary of the insecticide cannon sprayer; lines of black stars = exposed mosquito cages at 10, 30, 50 and 70 m from insecticide spraying; VC-1 and VC-2 = validation mosquito cages. (B) Department of Philosophy, Sapienza University = untreated site (same scale as A). Yellow dots = sticky traps. Background: OpenStreetMap data rendered with Landscape style, by opencyclemap.org, Map data OpenStreetMap contributors, CC BY-SA 2.0.

Experiments were carried out in two sites in central Rome at a 1.4 km distance from each other (Fig 1), where presence of *Aedes albopictus* was previously detected (BC, personal observation). The first was a ~ 40 h-area of the Sapienza University hospital "Policlinico Umberto I" (41°54'21'' N 12°30'41'' E), characterized by 14 m high XIX century buildings and large boulevards lined by Platanus trees and pedestrian walkways occasionally lined with bushes. The second site was ~2.5 h-area of the Department of Philosophy of Sapienza University (41°55'07'' N 12°31'01'' E) including a central 14 m high XIX century building and a neighbouring area characterized by tall trees, bushes, pedestrian walkways. While insecticide treatments were planned in the "Policlinico Umberto I" (hereafter treated site) during summer 2013 (see below), no treatments were performed in Department of Philosophy (hereafter untreated site).

### Insecticide treatments

Eight adulticide treatments (T1-T8) were performed in the treated area by qualified technicians from a private company (SOGEA s.r.l.) from June to October 2013, by spraying 1% water diluted PERMEX 22E (BlueLine; 92% permethrin + 1.64% tetramethrin + 6.4% piperonyl butoxide) with a cannon sprayer (series "ELITE 345–400" Spray Team snc) mounted on the back of a flatbed truck. The vehicle was driven at an average speed <20 km/h. Droplet size was set up at 50/60 μM. Spraying started around midnight and lasted for approximately 2 hours. Moreover, all the 227 rain catch basins (i.e. drain holes in paved streets sealed by grids) within the treated area (including empty basins to avoid risk of refilling in case of rain) were treated every two weeks from June to October by releasing tablets of an Insect-Growth-Regulators (IGR) which interferes with larval development and inhibits adult emergence (i.e. 0.5 gr pure Pyriproxyfen, PROXILAR, INDIA Industrie Chimiche).

### Cage experiments

Cylindrical cages (26 cm in diameter and 31 cm in height) lined with nylon tulle were manually built following Cooperband et al. (2007) [16]. Cages—containing Petri dishes with filter paper (Pall Corporation, 90 mm diameter) and *Ae*. *albopictus* adults (either 10 or 20 males and 10 or 20 females reared in the lab from wild collected eggs)—were positioned in the treated site at 1.5 m-height. During T2-T8 treatments, cages were located as follows: i) 12 cages along 3 roads (hereafter lines) at a 10, 30, 50 and 70 m distance from the crossroad to the itinerary of the cannon sprayer (hereafter exposed cages); ii) 2 validation cages within the treated site at 13 m (VC-1) and 41 m (VC-2) from the closest road where the cannon sprayer passed; and iii) 3 cages in the untreated site (hereafter control cages). Cages were located 1 hour before adulticide spraying and removed approximately 30 minutes after. The filter papers were immediately extracted from cages and introduced in a sealed glass vial for subsequent Gas-Chromatography Mass-Spectrometry (GC-MS) analysis. Adults were transferred to paper cups, provided with cotton pads soaked with 10% sucrose solution and brought to the lab. Mosquito mortality at 24 h post-exposure was recorded.

### Gas-Chromatography Mass-Spectrometry analysis

Gas-Chromatography Mass-Spectrometry analyses were carried out by Agilent 6850 II gas-chromatograph (GC) equipped with mass selective detector (MSD) Agilent mod. 5975C and capillary column Agilent HP-5 MS (60.0 m long x 0.25 mm i.d., 0.25 μm film thickness). The column operated at 60°C (hold 1 min) to 170°C (hold 0 min) at 10°C/min, then to 280°C (hold 5 min) at 4°C/min. The split/splitless injector was maintained at 250°C, and transfer line at 280°C. Helium was used as carrier gas at 1.4 mL/min. The MSD was used in the single ion monitoring mode (SIM). Insecticides were monitored by considering two ions for each compound, with the following masses (m/z): permethrin = 127 and 183; tetramethrin = 123 and 164; piperonyl butoxide = 119 and 176.

After withdrawal filters left in cages during the insecticide space-spraying were transferred in a cylinder and extracted 3 times with 5, 2.5 and 2.5 mL of hexane (Sigma-Aldrich, USA), respectively. The organic extracts were collected in a vial, sealed and stored at -20°C until analysis.

Analytical determinations were carried out by GC/MS with the external standard technique. Stock standard solutions of analysed insecticides at 100.0 ± 0.5 μg/mL were obtained by Ultra Scientific, USA. Working standard solutions (w.s.s.) for calibration were prepared daily and were obtained by diluting aliquots of the stock solution with hexane, to obtain working standard concentrations of 0.01, 0.05, 0.50, 1.00, 2.50, 5.00, and 10.00 μg/mL. All the glassware was in borosilicate class A. Calibration curves were obtained by injecting five 1 μL injections of each w.s.s. and calculating the average peak area for each different concentration. Linear responses were observed in the range of concentrations considered. Analytes concentrations were determined by three 1 μL injections of each sample extract, and average peak areas were considered for quantitation. Results were expressed as μg/cm^2^. Whole procedure blank tests were performed in order to assess the absence of any contamination occurring from reagents and materials. A solvent blank was analysed every five samples to check the response of chromatography.

### *Aedes albopictus* monitoring in the field

*Aedes albopictus* adult population monitoring was carried out from June 17^th^ to October 17^th^ 2013 in treated and untreated sites. Monitoring of adult populations was conducted by means of Sticky-Trap (ST) consisting in a water container similar to a commonly used ovitrap equipped with an internal structure lined with adhesive films to which the mosquitoes approaching the trap, either to lay eggs or to rest, remain stuck[17]. Sticky-Trap catches have been shown to be correlated with catches by ovitraps (i.e. the gold standard for *Ae*. *albopictus* monitoring), but collect eggs instead of adults [17] and have already been successfully exploited to assess the effectiveness of mosquito control interventions in Rome [14]. Sticky-Trap number and position was established subdividing an area within the treated site into a 24-cell grid and the untreated site into a 19-cell grid (each cell = 40 x 40 m) (Fig 1). One ST was located in each cell and equipped with sticky sheets and 500 ml tap water. On a weekly basis, mosquitoes stuck in ST were marked directly on sticky sheets after 72 hours (day-3); after additional 72 hours, STs were removed and stuck mosquitoes identified and counted under a binocular stereo microscope (day-6). No STs were left in the field at day-7, when insecticide spraying was performed if scheduled. Sticky-Traps equipped with freshly prepared sticky sheets were re-located in the same position at day-1 of each week. Water leftover was measured concomitantly to mosquito monitoring.

Temperature and rainfall data were obtained from “Roma Macao” weather-station at 300 m distance from the treated site (http://www.idrografico.roma.it/annali/).

### Statistical analysis

All analyses were carried out using R version 3.1.0 [18] and lme4, strucchange packages [19–21].

#### Assessment of effectiveness of insecticide spraying on caged *Aedes albopictus*

Effectiveness of single treatments on caged mosquitoes was computed by using the Henderson formula [22] adapted to the experimental protocol as follows:

$$\%Effectiveness=100\;*\;\left(1-\frac{mosquitoes\;treated\;after\;*\;mosquitoes\;untreated\;before}{mosquitoes\;treated\;before\;*\;mosquitoes\;untreated\;after}\right),$$ (1)

where *mosquitoes treated before* [*after*] are the mean numbers of alive mosquitoes in exposed cages before the treatment [after the treatment] and *mosquitoes untreated before* [*after*] are the corresponding mean numbers of alive mosquitoes in control cages.

Moreover, a first binomial Generalized Linear Mixed Model (GLMM-1) was carried out to test the effect of spraying treatments on caged mosquitoes. Date of treatment was introduced in the model as random effect to take into account the different environmental conditions exclusive of each treatment date (e.g. wind, climate). In addition, lines within date of treatments were modelled as nested random effect. Response variable was the proportion of dead mosquitoes out of the initial number in each cage, while explanatory variables were: i) exposure to insecticide treatments (exposed vs. control cages), ii) permethrin concentration in exposed cages as detected by GC-MS and iii) mosquito gender. All two-way interaction terms were included into the model.

A second binomial GLMM (GLMM-2) was carried out only for exposed cages to quantify the relationship between adult mortality and distance among cages and from insecticide spraying. As for GLMM-1, lines within date of treatments were modelled as nested random effect. Random structures were selected *a priori* [23,24]. Variance inflation factors and conditional boxplot were applied to assess collinearity. Finally, VC-1 and VC-2 (see above) were used to validate model prediction. For each cage we computed the adult mortality predicted by the model on the basis of the cage distance to the spraying. Then, given the initial number of mosquitoes in cages and using estimated mortality, we simulated the number of dead adults obtained by a random binomial sample for each of the seven treatments. Ten thousand random samples have been simulated resulting in the distribution of the expected mortality for each treatment. Observed mortality out of the 0.025 and 0.975 quantile of the expected distribution was considered statistically significant.

#### Assessment of effectiveness of insecticide sprayings on wild *Aedes albopictus* adults

Effectiveness of each treatment was computed by using Henderson formula (1) [22], where *mosquitoes treated before* [*after*] are the mean numbers of mosquitoes collected in all STs of the treated site in the 72 hours before [after] the treatment, while *mosquitoes untreated before* [*after*] are the corresponding (measured at same collection date) mean number of mosquitoes collected in all STs in the untreated site.

Linear Mixed Models (LMM-1 and LMM-2) were carried out to evaluate whether water leftover in STs could be a reliable proxy for eco-climatic conditions at finer scale (i.e. association between overall climatic conditions and ST exposure to sun-light) and whether it was different between treated and untreated sites. Model response variable was water leftover in each ST, while explanatory variables were average maximum daily temperature (for LMM-1) and daily rainfall (for LMM-2) recorded at closest weather station, sites (treated vs. untreated) and their interaction. Collection date and ST identification number were considered as random effects. The random structures were selected *a priori* [23,24].

A Poisson Generalized Linear Mixed Model (GLMM-3) was carried out to test whether *Ae*. *albopictus* abundance was different between sites, whether mosquito abundance at ST level was related to water leftover and whether this relationship changed between sites. Model response variable was mosquito count recorded in each ST, while explanatory variables were water leftover in ST, sites (treated vs. untreated) and their interaction. Collection date and ST identification number were considered as random effects. The random structures were selected *a priori* [23,24].

Change point analyses [25] were carried out to assess the impact of the control strategy adopted over time and to understand which drivers (i.e. insecticide treatments and/or eco-climatic conditions) were responsible for differences in observed mosquito abundance between treated and untreated sites. Time series of the average values of the mosquitoes collected during each collection date and of the corresponding water leftover in STs were compared between treated and untreated sites. Both series were pre-whitened by fitting them individually an autoregressive model ARIMA [26] to avoid distorted or misleading results as consequence of autocorrelation or common trends over time [27]. Afterwards, Pearson correlations between treated and untreated sites of ARIMA residuals for either mosquito or water leftover were computed. In order to evaluate whether correlation between treated and untreated sites changed during the season, correlation coefficients were computed by comparing 27 time series: the shortest series included 10 subsequent collection dates (from June 17^th^ to July 18^th^), while subsequent series were obtained by adding one collection at time until the end of the sampling (i.e. 36 collections). The temporal variation of the resulting 27 correlation coefficients was then compared between treated and untreated sites. Change point analyses were applied to detect abrupt changes in the mean of either mosquito and water leftover series of correlation coefficients, to estimate the number and location of changes of the mean of each series (see [28] for further details).

## Results

Results obtained for caged mosquitoes exposed to single insecticide treatments and results on the effectiveness of the overall control strategy adopted (i.e. adulticide sprayings and larvicide treatments of street catch basins) on the wild mosquito population are as follows (S1 Data).

### Effectiveness of insecticide sprayings on caged *Aedes albopictus* adults

The average effectiveness of the seven monitored insecticide sprayings assessed based on Henderson’s formula applied to caged mosquitoes was 77% (Confidence Interval: 93%—61%) at 10 m, 36% (CI: 49%—22%) at 30 m, 22% (CI: 35%—8%) at 50 m, 1% (CI: 2%— 0%) at 70 m from spraying (S1 Table). Restricting the analysis to cages located at ≤50 m distance from spraying (due to low mortality in the 70 m-distant cages), the average effectiveness of the treatments were as follows: T2 = 20.1%, T3 = 51.2%, T4 = 68.6%, T5 = 37.5%, T6 = 54.4%, T7 = 23.5%, T8 = 53.4%.

Results from the binomial GLMM-1 carried out to test the effectiveness of insecticide spraying on caged adult *Ae*. *albopictus* either exposed or not-exposed to the adulticide treatments indicated an overall higher mortality in exposed cages (Table 1; p = 0.002). No differences in mortality were detected between genders. As expected, permethrin detection was positively associated with mortality (S2 Table; p<0.001). However, mortality was observed also in cages where permethrin was not detected (concentration<0.0006 μg/cm^2^). Tetramethrin values were not taken into consideration for data elaboration as they were below the limit of detection of the analytical procedure.

**Table 1:** Binomial Generalized Linear Mixed Model of *Aedes albopictus* mortality in cages exposed and non-exposed to insecticide spraying.

GLMM-1 variables	Coeff.	SE^a^	z-value^b^	Pr(>|z|)^c^
Intercept	-3.642	0.506	-7.197	<0.001
Male	0.221	0.526	0.420	0.674
Perm. conc.	10.298	1.012	10.170	<0.001
Exposed	1.734	0.556	3.134	0.002
Male*Perm. conc.	-1.551	1.228	-1.265	0.206
Male* Exposed	-0.118	0.546	-0.216	0.829

Adult females in control cages set as reference (intercept). Number of observations = 184, groups = 28; treatment date = 7. Estimated random effect standard deviation for location within each treatment date = 0.9, for treatment date = 0.08.

^a^ Standard Error of parameter estimate

^b^ z-value estimate to standard error ratio

^c^ Pr(>|z|) statistic for z-value

*interaction term between independent variables

Moreover, the second binomial GLMM-2—carried out to assess mortality in cages at different distances from the insecticide spraying in the treated site (i.e. 10, 30, 50 and 70 m)—showed lower mortality at increasing distances (Estimated coefficient for Distance = -0.087; Z-value = -18.74; p<0.001).

**Fig 2:**
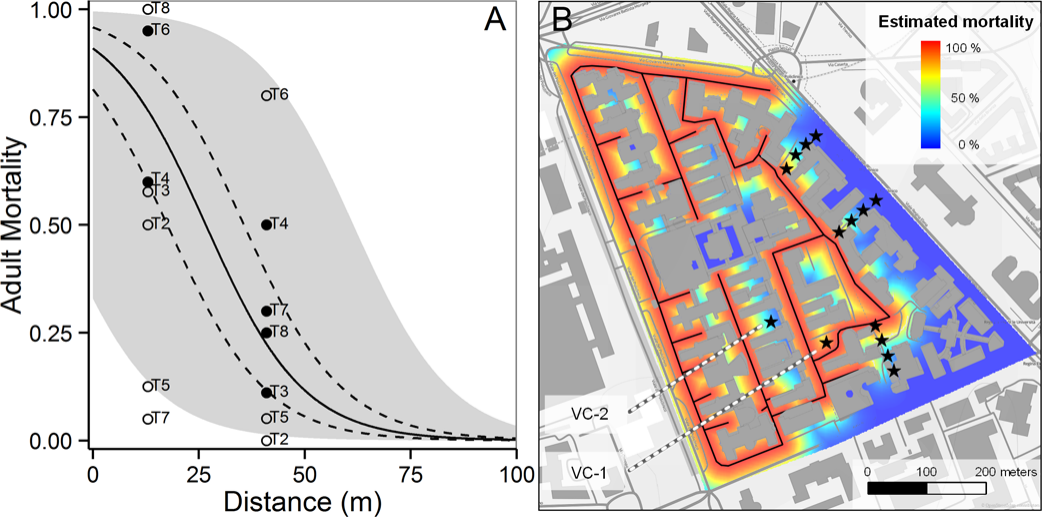
Expected effectiveness of insecticide sprayings in study area based on mortality observed in caged mosquitoes. (A) Expected *Aedes albopictus* adult mortality modelled as a function of the distance between the cages and the insecticide treatments (T2-T8), as predicted by GLMM-2. Central solid line = fitted values determined by the intercept and distance effect (fixed part); dashed lines = 95% confidence interval; grey area = uncertainty in predicted values due to variations in random terms (date and cage locations); circles = observed mortality values in validation cages (VC-1 and VC-2, 13m and 41m distant from spraying, respectively), either statistically different (empty circles) or non-statistically different (filled circles) from values simulated by GLMM-2. (B) Spatialized expected mosquito mortality modelled as a function of distance taken from binomial GLMM-2 result (fixed part) (central solid line in panel A). Lines of black stars = mosquito cages at 10, 30, 50 and 70 m from insecticide spraying. VC-1 and VC-2 = cages inside treated area used for GLMM-2 validation. Background: OpenStreetMap data rendered with Landscape style, by opencyclemap.org, Map data OpenStreetMap contributors, CC BY-SA 2.0.

Fig 2A shows expected adult mortality in treatment site modelled as a function of the distance between the cages and the insecticide spraying, as predicted by GLMM-2. Overall, adult mortality was predicted to be higher than 0.75 in 29% of the area not occupied by buildings, and higher than 0.50 in 41% of the same area (Fig 2B). Expected mortality obtained from GLMM-2 was validated by using mortality values observed in validation cages, located at 13m (VC-1) and 41m (VC-2) from spraying (Fig 2A and 2B). Mortality rates were extremely variable among treatments, ranging from 5 to 100% in VC-1 and from 0 to 80% in VC-2 (Fig 2A). In T7 observed mortality in VC-1 was even lower than in VC-2. Mortality in VC-1 (average observed value = 54%, predicted = 77%) and in VC-2 (average observed value = 29%, predicted = 22%) was outside the 0.025 and 0.975 quantile of the expected mortality distribution in 5 and 3 out of 7 monitored treatments, respectively (S1 Fig). Specifically, observed mortality was underestimated in 6 out of 8 of these cases (i.e. values<0.025), overestimated in 2 cases (i.e. values>0.975).

### Effectiveness of insecticide sprayings on wild *Aedes albopictus* adults

Henderson’s formula computed for each single insecticide spraying showed a mosquito female and male adult reduction only for 4 out of 8 treatments (i.e. T1 = 100%, T2 = 0%; T3 = 0%; T4 = 55.5%; T5 = 57.1%; T6 = 0%;T7 = 83.8%; T8 = 0%; S3 Table).

**Fig 3:**
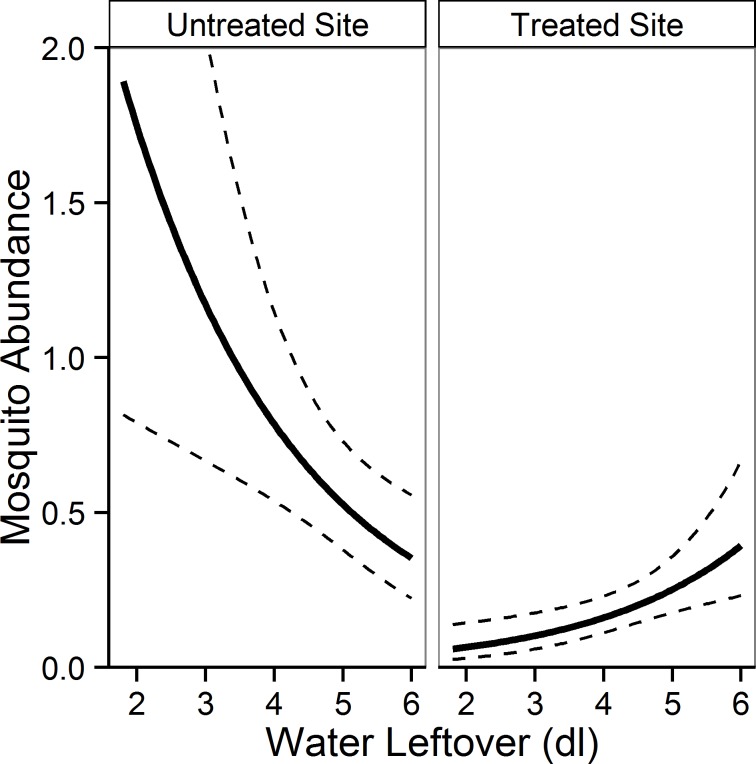
Plots of predicted mean *Aedes albopictus* abundance as a function of water leftover in sticky traps. Predictions in untreated and treated sites based on GLMM-3. X-axis = water leftover after 72 hours (5 dl initial water level; values>5 dl due to rainfall and/or artificial watering); Y-axis = predicted mean abundance in sticky traps; solid lines = predicted mean value; dashed lines = 95% confidence intervals.

However, the objective of the study was not only to evaluate effectiveness of single adulticide spraying, but also to assess the impact of the overall control strategy adopted (i.e. adulticide sprayings and larvicide treatments of street catch basins) taking into account the eco-climatic conditions in the two sites. In order to achieve this objective, water leftover inside ST was taken as a proxy for the specific eco-climatic conditions at ST level (i.e. association between overall climatic conditions and ST exposure to sun-light). This was based on LMM results showing a negative relationship between water leftover in ST and temperature (LMM-1; S4 Table; S2A Fig) and a positive relationship with rainfall (LMM-2; S5 Table; S2B Fig). Afterwards, measures of water leftover were included as explanatory variables in the Poisson GLMM-3 carried out to test how mosquito counts varied between treated and untreated sites. The result showed that mosquito counts were significantly higher in the untreated site (N in treated site = 231; N in untreated site = 552; p<0.001). However, while in the untreated site higher mosquito counts were observed in STs with lower values of water leftover, unexpectedly no relationship between mosquito counts and water leftover was observed in the treated site (Table 2; Fig 3).

**Table 2:** Poisson Generalized Linear Mixed Model of *Aedes albopictus* counts in sticky traps in insecticide treated and untreated sites.

GLMM3 variables	Coeff.	SE^a^	z-value^b^	Pr(>|z|)^c^
Intercept	1.358	0.659	2.062	0.0392
Treated	-4.976	0.777	-6.405	<0.0001
Water leftover	-0.400	0.134	-2.986	0.0029
Water leftover* Treated	0.848	0.167	5.091	<0.0001

The reference level is untreated site. Water leftover = water leftover in sticky trap (STs) during 72 hours. Number of observation = 1523, number of collections = 36, ST number = 43. Estimated random effect standard deviation for collection = 0.73, for ST = 0.42.

^a^ Standard Error of parameter estimate

^b^ z-value estimate to standard error ratio

^c^ Pr(>|z|) statistic for z-value

*interaction term between independent variables

**Fig 4:**
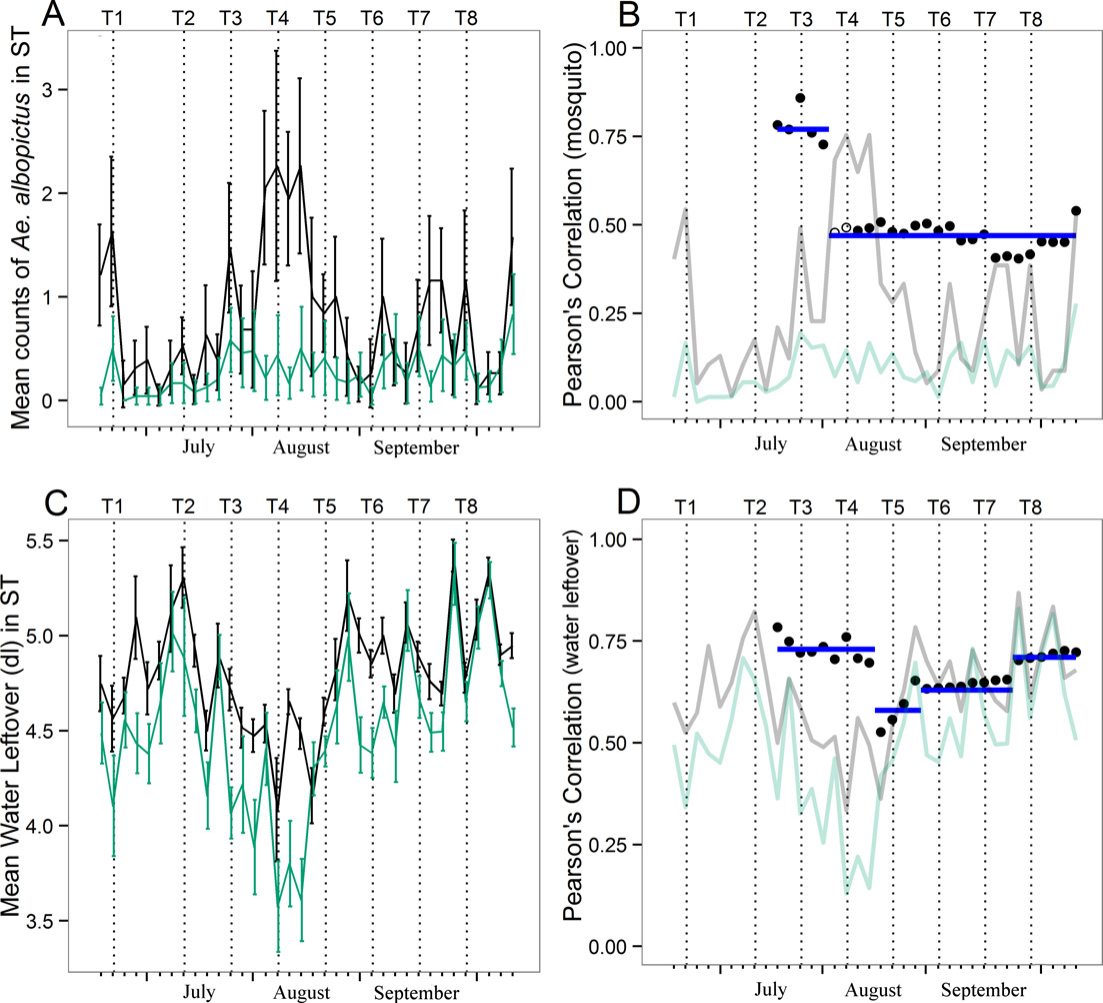
Change point analysis of *Aedes albopictus* abundance and of water leftover in study sites. (A) Seasonal pattern of mosquito abundance in insecticide-treated (green line, N = 24) and untreated (black line, N = 19) sites. (B) Correlation of residual of mosquito time series between treated and untreated sites. (C) Seasonal pattern of water leftover in sticky traps in insecticide-treated (green line, N = 24) and untreated (black line, N = 19) sites. (D) Correlation of residual of water leftover time series between treated and untreated sites. Solid circles = significant correlation estimates (p-value < 0.05). Empty circles = non-significant correlation estimates (p-value > 0.05). Blue horizontal line in B and D panels = fitted mean in each sequence; each break identifies a statistically significant change in mean. Vertical bars in A and C panels = 95% confidence intervals. X-axis = 2013 collection dates. Vertical dotted lines = dates of insecticide sprayings.

Finally, change point analysis was carried out to assess temporal variations of the impact of the control strategy adopted on the seasonal mosquito population dynamic (Fig 4A). Results showed a sharp decrease in correlation (Pearson’s coefficient from 0.77 to 0.47) between time series of adult mosquito mean counts in the treated and in the untreated site after T3 (collection 15, August 5^th^; Fig 4B). This change occurred when population in the untreated site was reaching its peak; afterwards, correlation between the two time series remained stable (Fig 4B). Change point analysis was also applied to water leftover between ST-time series in treated and untreated sites to understand whether eco-climatic conditions were a major determinant of differences observed in mosquito abundance between the two sites. Results showed a sharp decrease in correlation coefficients between the two sites at collection 19 (August 19^th^, after T4). Afterwards, an increase of correlation along the season was observed (Fig 4C and 4D).

## Discussion

The results obtained show that the effectiveness of sequential insecticide treatments on *Ae*. *albopictus* population dynamics may be assessed by coupling an intensive seasonal spatio-temporal monitoring of mosquito population dynamics and eco-climatic variations in treated vs untreated sites with the use of advanced statistical methods. These are necessary to disentangle the effect of the treatments from those of eco-climatic inter-site differences on mosquito population patterns. Thus, the proposed approach provides a reliable alternative to the need to have information on mosquito populations in treated and untreated sites in seasons/years before the effectiveness assessment. Moreover, it overcomes the difficulty in attributing inter-site differences in population patterns to the insecticide treatments rather than to site-specific eco-climatic variations. In fact, results of the temporal analysis showed that mosquito seasonal patterns were initially comparable in the two sites, diverged in the absence of diverging eco-climatic conditions and remained stable afterwards. This led us to attribute the lack of *Ae*. *albopictus* population expansion in the area of the main University hospital in Rome to the combined effect of multiple adulticide sprayings and regular larvicide treatments carried out during the whole season. In fact, a clear population expansion was observed in August in the untreated control site and it is known to typically occur in the same period in Rome[13,29]. The conclusion would have been very different if we would have speculated on the effectiveness of the treatments only based on Henderson’s formula results on caged mosquitoes and/or on field ST-collections before and after single sprayings in treated vs untreated sites. These results were variable and inconsistent. In the case of cage experiments, mortality was found negatively associated to distance from spraying and positively associated to Permethrin concentration, as expected. However, high variability in mortality was observed among cages within single treatments, as well as among treatments. Based on these results adult mortality was predicted to be higher than 50% only in 41% of the treated area. The high variability observed among caged mosquitoes was most likely due to variations in wind direction and/or strength (not measured), as suggested by the variable concentrations of Permethrin detected in cages. In the case of the assessment based on ST-collections of wild mosquitoes after single insecticide sprayings, results showed an adult reduction with respect to the untreated area only after 4 out of 8 treatments. This high variability could be at least partially due to the fact that we did not sample the sites immediately before and after the insecticide spraying (as implied by Henderson’s formula), but 3 days before and 3 days after each treatment, thus introducing the confounding factor of freshly adult emergence. Other factors intrinsic to field experiments may account for the inconsistency between results based on ST-collections and those based on cage experiments: e.g. i) “controls” are affected by the mosquito population dynamics in the field, but not in the cages; ii) mortality in cages is measured immediately after the treatment, thus reflecting the rapid knock-down effect, while assessment of treatment effectiveness in the field is based on ST-collection in the 72h following the treatment, thus reflecting both rapid knock-down and residual effect.

The methodological approach here proposed to assess the effectiveness of seasonal-long mosquito control strategies can be applied to assess the effectiveness of various control methods, under the assumption that the major forces determining mosquito population dynamics are eco-climatic factors. The approach relies on the possibility to compare mosquito population dynamics in treated and in untreated control sites by sticky trap collections, even in the absence of prior information on mosquito abundance and eco-climatic situation in these sites. In fact, water leftover in sticky trap was shown to be correlated with temperature (negatively) and rainfall (positively) and can thus be taken as a good proxy for the eco-climatic conditions at sticky trap level, synthetizing the association between overall climatic conditions and sticky trap exposure to sun-light. Notably, water leftover can be easily measured during routine sticky trap monitoring activities without significant additional efforts in terms of time and costs. This allowed us to compare with great resolution changes in correlation between time series of adult mosquito mean counts and seasonal changes of eco-climatic conditions in the treated and untreated sites and to reach the conclusion that the lack of *Ae*. *albopictus* population expansion in the treated site was due to the insecticide treatments rather than to eco-climatic factors. In theory, the methodological approach here proposed could be carried out by ovitrap collections, a widely used method to indirectly assess adult abundance. However, complete water evaporation is frequently observed in ovitraps after <3 days in very hot sites/seasons, such as in Rome in August (BC, personal observation), but not in STs which are supplied with a top lid. Moreover, ovitrap exploitation for assessing adult abundance based on number of collected eggs has been questioned [30]. On the other hand, it should be noted that monitoring STs is more laborious than ovitraps, due to the need to manipulate sticky-sheets.

Overall, our results suggest that the combined effect of adulticide sprayings and larvicide treatments carried out in the study site had an effect in reducing *Ae*. *albopictus* abundance–and probably its nuisance—during the seasonal peak of the species. Larvicide treatments seem to have had a major role in determining the observed lack in the mosquito population expansion, as suggested by the apparent low impact of single adulticide sprayings assessed based on caged and wild mosquitoes. The latter could be due, among other factors, to the spraying time (i.e. during the night to reduce human exposure to insecticides), when *Ae*. *albopictus* is believed to be less affected because of its diurnal activity. However, it should be mentioned that single night-time ULV adulticiding were shown to result in a significant percent of reduction in *Ae*. *albopictus* abundance in treated vs. untreated sites in the US [12,31].

Despite this study was carried out in a single location and replicated only once over one season, the conclusions are consistent with the preliminary indications on the effectiveness of a combined intervention based on IGR-treatments of catch basins and two insecticide sprayings carried out at the beginning of the major population expansion in Sapienza University campus in Rome [14]. This may suggest that interventions combining larvicide and adulticide treatments may have an effect even when sprayings are carried out only during the population expansion phase, thus allowing to reduce and optimize the use of insecticide ground spraying. Other studies are needed to confirm this hypothesis and to shed light on the relative contribution of larvicide and adulticide treatments.

It is relevant to remind that despite the overall agreement that integrated control strategies–mostly based on public education, source reduction and larvicide application, with insecticide spraying restricted to specific situations—are needed to significantly reduce *Ae*. *albopictus* abundance and associated nuisance [32], this is very rarely implemented. In fact, an integrated control strategy requires high level of public cooperation among local authorities, private companies, organized society, and communities and a continued support from both local authorities and communities. In practical terms, multiple calendar based adulticide sprayings associated to larvicide activities are offered by private companies to citizens in high *Ae*. *albopictus* infested areas, at least in Italy. Studies such as the present one are thus extremely important to provide information needed to optimize the planning of the treatments along the species reproductive season (for instance restricting insecticide sprayings to the beginning of the season, as suggested by present results) and more precisely assess their actual cost-benefits, also taking into account the environmental impact of adulticide ground spraying.

## Supporting Information

S1 Table Effectiveness (%) of single insecticide sprayings on mosquitoes in exposed cages based on Henderson’s formula. Positive values indicate a reduction in treated site after adjusting with control site reduction. Row = Road along which cages were located at various distance from insecticide spraying (see Fig 1).
(PDF)

S2 Table Concentration of Permethrin detected in each exposed cage after single insecticide sprayings. nd = Permethrin concentration under detection threshold (< 0.0006 μg/cm^2^); na = no exposed cages available. Row = Road along which cages were located at various distance from insecticide spraying (see Fig 1).
(PDF)

S3 Table Effectiveness (%) of single insecticide sprayings on wild mosquitoes based on Henderson’s formula. Positive Effectiveness values indicate a reduction in treated site after adjusting with control site reduction. Zero percentage values indicate a minor reduction in treated site compared to control site or no reduction post treatment at all. ST = Number of active STs pre/post insecticide treatment.
(PDF)

S4 Table Linear Mixed Model of water leftover in sticky traps located in treated and untreated site as a function of temperature. The reference level is untreated site. Number of observation = 1523, number of collections = 36, number of trap = 43. Estimated random effect standard deviation: collection = 0.27, trap = 0.18
(PDF)

S5 Table Linear Mixed Model of water leftover in sticky traps located in treated and untreated site as a function of rainfall. The reference level is untreated site. Number of observation = 1523, number of collections = 36, number of trap = 43. Estimated random effect standard deviation: collection = 0.29, trap = 0.18.
(PDF)

S1 Fig Distribution of expected *Aedes albopictus* mortality in validation cages after adulticide treatments. A = VC-1 (13 m distant from spraying); B = VC-2 (41 m distant). N = number of initial mosquito adults in cages in each treatment (T2-T8). Dashed black line = observed mosquito mortality (values reported in each graph); red vertical line at distribution mean = predicted mortality based on GLMM-2 (VC-1: 77%, VC-2: 22%); red segment at the bottom = 95% credible interval. X-axis = mosquito mortality; Y-axis = probability density.
(PDF)

S2 Fig **Result of Linear Mixed Model for relationship between water leftover in sticky trap and temperature (A) or rainfall (B) in treated and untreated site.** Initial values of water leftover = 5 dl; values >5 dl are due to rainfall or artificial watering. Lines = predicted mean value of water leftover; dashed line = 95% confidence intervals. Green line = treated site; black line = untreated site.
(PDF)

S1 Data Cage mosquitos = mortality on caged *Aedes albopictus* adults; Wild mosquitos = wild *Aedes albopictus* adults collected by sticky traps in treated and untreated sites. (XLSX)
