# Supplementary material for: Assessment of the Effectiveness of a Seasonal-Long Insecticide-Based Control Strategy against Aedes albopictus Nuisance in an Urban Area
Source: PLoS Negl Trop Dis. 2016 Mar 3;10(3):e0004463. doi: 10.1371/journal.pntd.0004463 (PMC4777573; doi:10.1371/journal.pntd.0004463)
Supplement: S1 Table — Positive values indicate a reduction in treated site after adjusting with control site reduction. Row = Road along which cages were located at various distance from insecticide spraying (see Fig 1). (PDF) [file pntd.0004463.s001.pdf]

Supplementary Tables

Table S1. Effectiveness (%) of single insecticide sprayings on caged mosquitoes based on Henderson's formula.

		Distance from insecticide spraying				
**Treatment**	**Row**	**10 m**	**30 m**	**50 m**	**70 m**	**Total**
**T2**	1	37.93	1.72	12.07	-3.45	12.07
	2	1.72	12.07	1.72	-3.45	3.02
	3	100.00	1.72	1.72	6.90	27.59
	Total	46.55	5.17	5.17	0.00	14.22
**T3**	1	94.74	15.79	10.53	-	40.35
	2	100.00	31.58	21.05	-	50.88
	3	100.00	73.68	57.89	-	77.19
	Total	98.25	40.35	29.82	-	56.14
**T4**	1	100.00	57.45	62.77	-	73.40
	2	100.00	68.09	46.81	-	71.63
	3	100.00	57.45	41.49	-	66.31
	Total	100.00	60.99	50.35	-	70.45
**T5**	1	1.77	-0.88	9.73	7.08	4.42
	2	94.69	52.21	28.32	1.77	44.25
	3	86.73	81.42	49.56	1.77	54.87
	Total	61.06	44.25	29.20	3.54	34.51
**T6**	1	94.83	100.00	94.83	1.72	72.84
	2	100.00	12.07	-3.45	1.72	27.59
	3	6.90	12.07	6.90	1.72	6.90
	Total	67.24	41.38	32.76	1.72	35.78
**T7**	1	-1.79	8.93	8.93	3.57	4.91
	2	100.00	-1.79	-7.14	-1.79	22.32
	3	100.00	35.71	-7.14	-1.79	31.70
	Total	66.07	14.29	-1.79	0.00	19.64
**T8**	1	100.00	64.10	7.69	-2.56	42.31
	2	100.00	23.08	12.82	-2.56	33.33
	3	100.00	58.97	-2.56	2.56	39.74
	Total	100.00	48.72	5.98	-0.85	38.46

Positive values indicate a reduction in treated site after adjusting with control site reduction. Row=Road along which cages were located at various distance from insecticide spraying (see Figure 1).
