# Supplementary material for: Assessment of the Effectiveness of a Seasonal-Long Insecticide-Based Control Strategy against Aedes albopictus Nuisance in an Urban Area
Source: PLoS Negl Trop Dis. 2016 Mar 3;10(3):e0004463. doi: 10.1371/journal.pntd.0004463 (PMC4777573; doi:10.1371/journal.pntd.0004463)
Supplement: S2 Table — nd = Permethrin concentration under detection threshold (< 0.0006 μg/cm2); na = no exposed cages available. Row = Road along which cages were located at various distance from insecticide spraying (see Fig 1). (PDF) [file pntd.0004463.s002.pdf]

Table S2. Concentration of Permethrin detected in each cage after single insecticide sprayings.

		Distance from insecticide spraying				
**Treatment**	**Row**	**10 m**	**30 m**	**50 m**	**70 m**	**Total**
**T2**	1	0.001682827	nd	nd	nd	0.001682827
	2	nd	nd	0.001359008	nd	0.001359008
	3	0.06454743	0.000794418	nd	nd	0.065341848
	Total	0.066230257	0.000794418	0.001359008	nd	0.068383683
**T3**	1	0.003272307	0.001362227	nd	na	0.004634534
	2	0.085726332	nd	nd	na	0.085726332
	3	0.021510445	0.001600424	0.002191409	na	0.025302278
	Total	0.110509084	0.002962651	0.002191409	na	0.115663144
**T4**	1	0.002228104	0.001169095	0.001218022	na	0.004615221
	2	0.022383404	0.126931778	0.001490339	na	0.150805521
	3	0.036653958	0.003057286	0.003838829	na	0.043550073
	Total	0.061265466	0.131158159	0.00654719	na	0.198970815
**T5**	1	nd	0.000733259	nd	nd	0.000733259
	2	0.010346104	nd	nd	nd	0.010346104
	3	0.003798915	0.001121456	nd	nd	0.004920371
	Total	0.014145019	0.001854715	nd	nd	0.015999734
**T6**	1	0.017361961	0.002483039	nd	0.000653431	0.020498431
	2	0.003509216	nd	nd	nd	0.003509216
	3	nd	nd	nd	nd	nd
	Total	0.020871177	0.002483039	nd	0.000653431	0.024007647
**T7**	1	nd	nd	nd	nd	nd
	2	0.031839811	nd	nd	nd	0.031839811
	3	0.006144186	0.002258362	nd	nd	0.008402548
	Total	0.037983997	0.002258362	nd	nd	0.040242359
**T8**	1	0.008507483	0.000885834	nd	nd	0.009393317
	2	0.003855567	nd	nd	nd	0.003855567
	3	nd	nd	nd	nd	nd
	Total	0.01236305	0.000885834	nd	nd	0.013248884

nd = Permethrin concentration under detection threshold (< 0.0006 µg/cm^2); na = no exposed cages available.
Row=Road along which cages were located at various distance from insecticide spraying (see Figure 1).
