# Supplementary material for: Assessment of the Effectiveness of a Seasonal-Long Insecticide-Based Control Strategy against Aedes albopictus Nuisance in an Urban Area
Source: PLoS Negl Trop Dis. 2016 Mar 3;10(3):e0004463. doi: 10.1371/journal.pntd.0004463 (PMC4777573; doi:10.1371/journal.pntd.0004463)
Supplement: S3 Table — Positive Effectiveness values indicate a reduction in treated site after adjusting with control site reduction. Zero percentage values indicate a minor reduction in treated site compared to control site or no reduction post treatment at all. ST = Number of active STs pre/post insecticide treatment. (PDF) [file pntd.0004463.s003.pdf]

Table S3. Effectiveness (%) of single insecticide sprayings on wild mosquitoes based on Henderson's formula.

	**Treated site**			**Control site**			
Treatment	ST	Pre-treatment Mean (± SE)	Post-treatment Mean (± SE)	ST	Pre-treatment Mean (± SE)	Post-treatment Mean (± SE)	Effectiveness (%)
**T1**	24/23	0.50 ± 0.16	0.00 ± 0.00	19/19	1.63 ± 0.37	0.16 ± 0.12	100%
**T2**	24/24	0.17 ± 0.10	0.08 ± 0.06	19/19	0.53 ± 0.14	0.11 ± 0.07	0%
**T3**	24/24	0.58 ± 0.16	0.46 ± 0.17	19/19	1.47 ± 0.32	0.68 ± 0.22	0%
**T4**	23/24	0.43 ± 0.20	0.17 ± 0.08	19/19	2.26 ± 0.57	1.95 ± 0.33	55.5%
**T5**	22/24	0.41 ± 0.18	0.21 ± 0.10	19/19	0.84 ± 0.19	1.00 ± 0.30	57.1%
**T6**	24/24	0.04 ± 0.04	0.38 ± 0.13	19/19	0.26 ± 0.17	1.00 ± 0.29	0%
**T7**	21/22	0.52 ± 0.15	0.14 ± 0.07	18/19	0.72 ± 0.23	1.16 ± 0.32	83.8%
**T8**	19/24	0.47 ± 0.14	0.12 ± 0.07	19/19	1.16 ± 0.34	0.11 ± 0.07	0%

Positive Effectiveness values indicate a reduction in treated site after adjusting with control site reduction. Zero percentage values indicate a minor reduction in treated site compared to control site or no reduction post treatment at all. ST = Number of active STs pre/post insecticide treatment.
