# Supplementary material for: Assessment of the Effectiveness of a Seasonal-Long Insecticide-Based Control Strategy against Aedes albopictus Nuisance in an Urban Area
Source: PLoS Negl Trop Dis. 2016 Mar 3;10(3):e0004463. doi: 10.1371/journal.pntd.0004463 (PMC4777573; doi:10.1371/journal.pntd.0004463)
Supplement: S4 Table — The reference level is untreated site. Number of observation = 1523, number of collections = 36, number of trap = 43. Estimated random effect standard deviation: collection = 0.27, trap = 0.18 (PDF) [file pntd.0004463.s004.pdf]

**Table S4. Linear Mixed Model of water leftover in sticky traps located in treated and untreated site as a function of temperature.**

| **LMM-1** | **Coeff.** | **Std. Error** | **t-value** | **Pr(>\|t\|)** |
|---|---|---|---|---|
| Intercept | 5.141 | 0.101 | 47.685 | <0.0001 |
| Treated | -0.149 | 0.066 | -2.252 | 0.027 |
| Temperature | -0.044 | 0.012 | -3.768 | 0.0005 |
| Temperature*Treated | -0.020 | 0.005 | -4.616 | <0.0001 |

The reference level is untreated site. Number of observation = 1523, number of collections = 36, number of trap = 43. Estimated random effect standard deviation: collection = 0.27, trap = 0.18
