# Supplementary material for: Assessment of the Effectiveness of a Seasonal-Long Insecticide-Based Control Strategy against Aedes albopictus Nuisance in an Urban Area
Source: PLoS Negl Trop Dis. 2016 Mar 3;10(3):e0004463. doi: 10.1371/journal.pntd.0004463 (PMC4777573; doi:10.1371/journal.pntd.0004463)
Supplement: S5 Table — The reference level is untreated site. Number of observation = 1523, number of collections = 36, number of trap = 43. Estimated random effect standard deviation: collection = 0.29, trap = 0.18. (PDF) [file pntd.0004463.s005.pdf]

Table S5. Linear Mixed Model of water leftover in sticky traps located in treated and untreated site as a function of rainfall.

**LMM-2**	**Coeff.**	**Std. Error**	**t-value**	**Pr(>|t|)**
Intercept	4.725	0.070	67.227	<0.0001
Treated	-0.348	0.058	-5.998	<0.0001
Mm of rain	0.011	0.004	2.994	0.0049
Mm of rain *Treated	0.006	0.001	4.744	<0.0001

The reference level is untreated site. Number of observation = 1523, number of collections = 36, number of trap = 43. Estimated random effect standard deviation: collection = 0.29, trap = 0.18.
