# Supplementary material for: Assessment of the Effectiveness of a Seasonal-Long Insecticide-Based Control Strategy against Aedes albopictus Nuisance in an Urban Area
Source: PLoS Negl Trop Dis. 2016 Mar 3;10(3):e0004463. doi: 10.1371/journal.pntd.0004463 (PMC4777573; doi:10.1371/journal.pntd.0004463)
Supplement: S1 Fig — A = VC-1 (13 m distant from spraying); B = VC-2 (41 m distant). N = number of initial mosquito adults in cages in each treatment (T2-T8). Dashed black line = observed mosquito mortality (values reported in each graph); red vertical line at distribution mean = predicted mortality based on GLMM-2 (VC-1: 77%, VC-2: 22%); red segment at the bottom = 95% credible interval. X-axis = mosquito mortality; Y-axis = probability density. (PDF) [file pntd.0004463.s006.pdf]

Supplementary Figures

Figure S1. Distribution of expected *Aedes albopictus* mortality in validation cages (VC) after adulticide treatments.

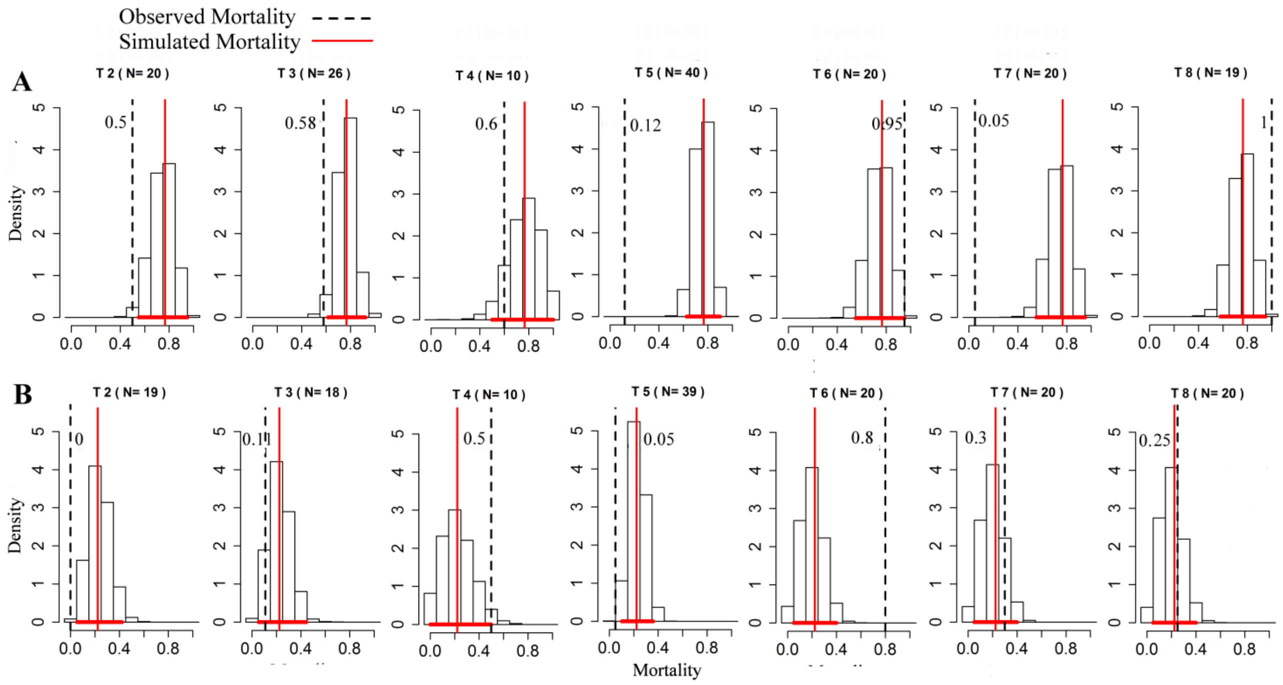

A=VC-1 (13 m distant from spraying); B=VC-2 (41 m distant). N=number of initial mosquito adults in cages in each treatment (T2-T8). Dashed black line=observed mosquito mortality (values reported in each graph); red vertical line at distribution mean=predicted mortality based on GLMM-2 (VC-1: 77%, VC-2: 22%); red segment at the bottom=95% credible interval. X-axis=mosquito mortality; Y-axis=probability density.
