# Supplementary material for: Assessment of the Effectiveness of a Seasonal-Long Insecticide-Based Control Strategy against Aedes albopictus Nuisance in an Urban Area
Source: PLoS Negl Trop Dis. 2016 Mar 3;10(3):e0004463. doi: 10.1371/journal.pntd.0004463 (PMC4777573; doi:10.1371/journal.pntd.0004463)
Supplement: S2 Fig — Result of Linear Mixed Model for relationship between water leftover in sticky trap and temperature (A) or rainfall (B) in treated and untreated site. Initial values of water leftover = 5 dl; values >5 dl are due to rainfall or artificial watering. Lines = predicted mean value of water leftover; dashed line = 95% confidence intervals. Green line = treated site; black line = untreated site. (PDF) [file pntd.0004463.s007.pdf]

Figure S2. Result of Linear Mixed Model for relationship between water leftover in sticky trap and temperature (A) or rainfall (B) in treated and untreated site.

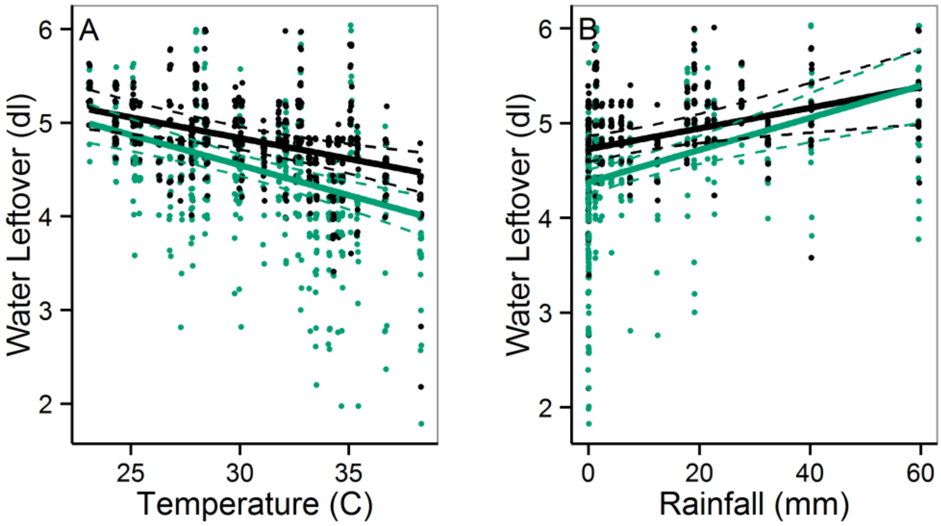

Initial values of water leftover= 5 dl; values >5 dl are due to rainfall or artificial watering. Lines=predicted mean value of water leftover; dashed line=95% confidence intervals. Green =treated site; black =untreated site.
